# *QuickStats*: Percentage* of All Visits by Patients Aged ≥65 Years to Office-Based Physicians^†^ Made by Patients with Hypertension,^§^ by Sex and Metropolitan Statistical Area (MSA)^¶^ — National Ambulatory Medical Care Survey, United States, 2012–2015

**DOI:** 10.15585/mmwr.mm6640a9

**Published:** 2017-10-13

**Figure:**
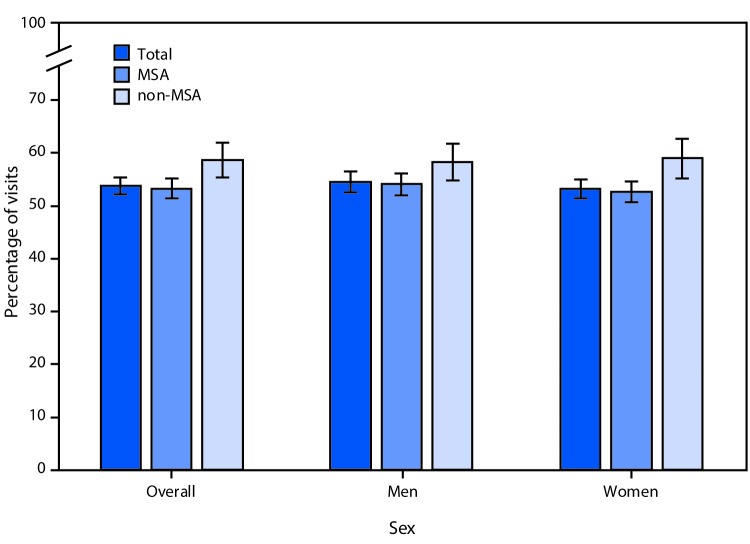
During 2012–2015, patients aged ≥65 years with hypertension documented in the medical record accounted for 54% of all office-based physician visits made by patients aged ≥65 years, with a higher percentage of visits in non-MSAs (59%) than MSAs (53%). Among women, the percentage of visits was also higher in non-MSAs than in MSAs (59% versus 53%). The difference among men was not statistically significant.

**Source:** National Center for Health Statistics, National Ambulatory Medical Care Survey, 2012–2015. https://www.cdc.gov/nchs/ahcd/ahcd_questionnaires.htm.

